# Psychometric properties of the Death Anxiety Scale for adult chronic patients

**DOI:** 10.47626/2237-6089-2023-0630

**Published:** 2025-02-27

**Authors:** Ximena Palacios-Espinosa, Ricardo Sánchez Pedraza, Ana-María Gómez-Carvajal, Juan Sebastián Botero-Meneses, Diana María Escallón, Diego Armando Leal

**Affiliations:** 1 Universidad del Rosario School of Medicine and Health Science People, Family and Society Research Group Bogotá Colombia Universidad del Rosario, School of Medicine and Health Science, People, Family and Society Research Group, Bogotá, Colombia.; 2 Instituto de Investigaciones Clínicas Facultad de Medicina Universidad Nacional de Colombia Bogotá Colombia Instituto de Investigaciones Clínicas, Facultad de Medicina, Universidad Nacional de Colombia, Bogotá, Colombia.; 3 Universidad del Rosario School of Medicine and Health Sciences, Neuroscience Center Neurovitae Bogotá Colombia Universidad del Rosario, School of Medicine and Health Sciences, Neuroscience Center Neurovitae-UR, Neuroscience Research Group NEUROS, Bogotá, Colombia.; 4 Hospital Universitario Méderi Bogotá Colombia Hospital Universitario Méderi, Bogotá, Colombia.; 5 Universidad del Rosario School of Medicine and Health Science Bogotá Colombia Universidad del Rosario, School of Medicine and Health Science, Bogotá, Colombia.

**Keywords:** Chronic disease, death anxiety, instrumental case study, psychometric properties

## Abstract

**Objectives:**

Death anxiety (DA) is a predictor of exacerbation of both physical and psychological symptoms of chronic diseases. Therefore, having short and easy-to-apply instruments to assess the presence of DA and adopting a multidisciplinary approach to address it are important. This study analyzes the psychometric properties of the Death Anxiety Scale (DAS), originally developed by Donald Templer, in a Colombian population of adult patients diagnosed with a chronic disease.

**Methods:**

The original instrument was linguistically, conceptually, and culturally adapted to Colombian Spanish to be subsequently administered to 301 adult patients with chronic diseases.

**Results:**

The exploratory factor analysis revealed a three-factor structure, which explained 47% of variance. Internal consistency was demonstrated (Cronbach’s alpha: 0.71; McDonald’s omega: 0.76; Guttman’s lambda 6 [G6]: 0.74; greatest lower bound: 0.54). A correlation coefficient of 0.64 was found between the total score of the DAS and the Beck Anxiety Inventory (BAI).

**Conclusion:**

When comparing the results with the versions of the DAS in Spanish from Mexico and Spain, variability in the psychometric properties was observed. Language cannot therefore be assumed to be a guarantee of the reliability and validity of the instrument.

## Introduction

Death anxiety (DA) is a universal phenomenon^1^ defined as an “emotional reaction produced by the perception of real or imaginary signs of danger or threat to one’s own existence, that may be triggered by environmental, situational, or dispositional stimuli, associated with one’s own or other people’s death.”^2^ It includes negative emotional reactions,^3,4^ nonspecific feelings of discomfort or unease^3^ caused by the individual’s anticipation of a state wherein the self does not exist,^4^ and “apprehension about the idea of their demise, the ‘nonbeing,’ and the uncertainty of what awaits us (or not) after death.”^3^ The stimuli triggering DA can be either learned or innate (thoughts or images).^5^ Nowadays, DA is acknowledged as a “multidimensional construct related to the fear or anxiety caused by the anticipation and awareness of the reality of death or dying, including emotional, cognitive, and motivational components that vary according to the developmental stage and the sociocultural context.”^5^ Cultural history, personal background, and ways of coping with separation and changes are factors linked to DA.^3^

The empirical background indicates that DA tends to be present in people suffering from chronic diseases, such as cancer,^6-9^ human immunodeficiency virus/acquired immunodeficiency syndrome (HIV/AIDS),^10^ cardiovascular conditions,^8,11,12^ chronic obstructive pulmonary disease (COPD),^13,14^ or diabetes mellitus,^8,15^ interfering with their health-related quality of life^10,16,17^ and imposing an additional burden affecting their experience with the disease and their coping skills. Assessing DA experienced by patients with chronic diseases is useful to help them to adjust after identifying the disease and taking prompt action to treat it. It is worth considering that such patients’ medical conditions may include tiredness, dyspnea, pain, and distress, among other signs and symptoms that interfere with their willingness to participate in and tolerate extensive assessment processes. It would therefore be advisable to have short and effective instruments to measure DA. The Death Anxiety Scale (DAS) was developed by Donald Templer and is a short, valid, and reliable 15-item instrument that is widely used and can be self-administered.^18^ Templer did not initially report the instrument’s factor structure, although it was later used in other studies that did describe it, obtaining varying results (Supplementary Table S1).

Overall, in addition to its widespread use and its translation into 26 languages, the DAS is easy to understand. Although versions in Spanish from Spain^19^ and Mexico^20^ are already available, these linguistic varieties of the language may make them difficult for other groups of Spanish speakers. Moreover, as far as we know, there is no version of this scale that has been translated into Colombian Spanish. Furthermore, its psychometric properties in a Colombian population of chronic patients are unknown. In this context, this study assesses the psychometric properties of the DAS, originally developed by Donald Templer, in a Colombian population of adult patients diagnosed with chronic disease.

## Materials and methods

### Participants

This study recruited adults who were diagnosed with a chronic illness, either receiving outpatient care or treated at a private hospitals in the city of Bogotá (Colombia), enrolled in the health and social security, once they had given informed consent to participation. Patients in altered states of consciousness or those feeling unwell at the moment of evaluation were excluded from the study. Based on these criteria, 301 individuals were sampled using a non-probabilistic sequential convenience method. Twelve patients decided not to take part in the study, and two were in severe pain at the time of the assessment, which prevented them from participating.

### Instruments

#### Death Anxiety Scale (DAS)

The DAS comprises 15 items on a dichotomous scale, where nine items are true and six are false. The interviewee is asked to mark their response considering whether each statement is true or false (always or most of the time). Each item is scored with values of 0 or 1, such that the score may range from 0 to 15, where a score closer to 0 represents a lower DA, while scores closer to 15 indicate greater DA. Test-retest reliability was 0.83, and the Kuder-Richardson Formula 20 value was 0.76, indicating the instrument’s internal reliability. We applied the cross-culturally adapted Colombian Spanish version in this study (Table 1).

**Table 1:** Death Anxiety Scale (DAS) Colombian Spanish version

Items		
1. Tengo mucho miedo de morir.	Verdadero	Falso
2. La idea de la muerte casi nunca entra en mi mente.	Verdadero	Falso
3. No me pone nervioso que la gente hable sobre la muerte.	Verdadero	Falso
4. Me aterra pensar que me tengan que operar.	Verdadero	Falso
5. No tengo ningún temor de morir.	Verdadero	Falso
6. No le tengo especial miedo a tener cáncer.	Verdadero	Falso
7. No me molesta la idea de la muerte.	Verdadero	Falso
8. Con frecuencia me siento preocupado(a) por lo rápido que pasa el tiempo.	Verdadero	Falso
9. Me da miedo morir dolorosamente.	Verdadero	Falso
10. Me perturba mucho el tema de la vida después de la muerte.	Verdadero	Falso
11. Tengo mucho miedo de tener un infarto.	Verdadero	Falso
12. Con frecuencia pienso en lo corta que es la vida.	Verdadero	Falso
13. Me estremezco cuando escucho a la gente hablar de una tercera guerra mundial.	Verdadero	Falso
14. Ver un cadáver es espantoso para mí.	Verdadero	Falso
15. Siento que no tengo nada que temer con respecto al futuro	Verdadero	Falso

The instructions for scale administration are as follows: Por favor responda las siguientes 15 preguntas. Si para usted la afirmación es verdadera, SIEMPRE O LA MAYORÍA DE VECES, marque VERDADERO. Si para usted la afirmación es falsa, SIEMPRE O LA MAYORÍA DE VECES, marque FALSO.

#### Beck Anxiety Inventory (BAI) (Beck & Steer – Spanish version)

Used to assess the discriminant validity of the DAS, the BAI measures general clinical anxiety. It includes 21 symptoms that are scored on a scale from 0 to 3, according to their presence. Scores between 0 and 63 are thus obtained, ranking the degree of anxiety as minimum, mild, moderate, or severe. This study used the Spanish version developed by Sanz (internal consistency: 0.90), currently published by the R&D Department of Pearson Clinical and Talent Assessment.^21^

#### Death Anxiety Inventory (DAI) (Tomás-Sábado & Gómez-Benito)

This instrument contains 20 items answered on a six-point Likert ranking scale (Cronbach’s alpha: 0.90; test-retest correlation: 0.94). The DAI is positively correlated with the DAS (0.79), and comprises five factors: 1) externally generated DA; 2) meaning and acceptance of death; 3) thoughts about death; 4) life after death; and 5) brevity of life.^22^

## Procedure

An independent Research Ethics Committee evaluated and approved the study, authorizing use of oral informed consent (code: DVO005-1-181-CEI903). All participants gave their consent to participation.

The study took place in three phases: translation and cross-cultural adaptation of the DAS, pilot testing, and application for validation.

## Statistical analysis

Conventional statistical tools were used for the descriptive component in accordance with the characteristics of the variables: means and standard deviations (SD) for continuous variables and percentages for categorical variables.

An exploratory factor analysis was conducted with the data from the sample of 301 patients to establish the instrument’s factor structure and explore the distribution of items across these factors or domains. Given the characteristic measurement method of the items, a tetrachoric correlation matrix was used for this analysis. The adequacy for factorization of the correlation matrix was checked using the Barlett and Kaiser-Meyer-Olkin (KMO) tests. The number of factors to assess was determined using the optimum coordinate method, by evaluating the characteristics of the sediment graphs. Additionally, orthogonal and oblique rotations were performed to select the factor structure with greatest interpretability.

For the exploratory factor analysis, the robust weighted least squares method was used for the data from the sample of 301 patients, given the dichotomous nature of the items. The model’s fit was deemed adequate provided the following cutoff values for these indices were achieved: ratio χ^2^/degrees of freedom (df) < 3; non-normed fit index (NNFI) > 0.9; root mean square error of approximation (RMSEA) < 0.08; goodness-of-fit index (GFI) > 0.9; comparative fit index (CFI) > 0.9; and standardized root mean squared residual (SRMR) < 0.08.

The instrument’s reliability, assessed based on internal consistency values, was measured using Cronbach’s alpha, McDonald’s omega, Guttman’s lambda, and the greatest lower bound (Supplementary Table S2).

Concurrent criterion validity was evaluated in 241 patients, estimating Pearson’s coefficients for the correlations between the total scores of the DAS and DAI scales and between the scores for the DAS domains and the overall score of the DAI scale.

To assess test-retest reliability, means were compared using the signed rank-sum tests. Additionally, Lin’s concordance correlation coefficients were estimated, and Bland-Altman limits of agreement plots were evaluated. All statistical analyses were conducted using R statistical software; 5% levels of significance and the two-tailed hypothesis were used for hypothesis tests (Supplementary Table S3).

For Rasch analysis, considering the characteristics of the items (yes-no responses) a dichotomous Rasch model was estimated using Winsteps (version 5.2.2). Two Rasch assumptions (unidimensionality and local independence) were tested. For unidimensionality, we used a principal component analysis of residuals (PCAR) as well as estimation of mean-squared (MNSQ) infit and outfit values. Unidimensionality is supported by eigenvalues < 2 for the first contrast and MNSQ infit values of 0.6-1.5.

Unidimensionality is questionable when considering eigenvalues > 3 for all contrasts and infit-outfit estimators that fall outside of the 0.7-1.3 range. Another criterion for establishing unidimensionality is based on the proportion of explained variance using the following measurements: variance explained should be > 20% (ideally 40%), whilst the variance that is not explained by the first contrast should not be greater than 15%.^23^ Local independence assumes that responses to an item are independent from responses to another item because the effect of causal dimension has been controlled for. This assumption was evaluated by calculating the correlations of standardized residuals. Considering item and person measures, we described them on a common, linear interval-level scale, using the logit (log odds). To evaluate item and person fit, the criteria of infit MNSQ (sensitive to unexpected responses close to the persons’ skill level) and outfit MNSQ (which prioritizes items that are far from the persons’ skill level) were used. Item difficulty was analyzed using a Wright map; this tool allows visual evaluation of where the items are located on a continuum of difficulty. Reliability was evaluated using person and item reliability indexes, and person and item separation indexes. The person separation index evaluates the extent to which the instrument is capable of distinguishing between two groups of subjects. The item separation index enables verification of whether a larger sample size would be required to confirm the separation of items (between lower or higher difficulty). Separation indexes > 1.5 and reliability coefficients > 0.7 represent acceptable levels.^24,25^

## Results

### Cross-cultural adaptation

Translation/back-translation processes were performed to find a linguistically, conceptually, and culturally equivalent translation. Initially, two bilingual health professionals translated the original scale into Spanish. These translations were sent to two bilingual healthcare providers, who each back-translated one of them into English, being blinded to the original items of the scale. Next, a committee of experts assessed the equivalence of the content between the test items in the English and Spanish versions. Each translation was considered in terms of aspects associated with meaning, expression, and grammar, and all items were included in a single translated version. Finally, an expert group was asked to compare the back-translation against the original version, item by item, thus resulting in the final version of the 15-item instrument. This version was administered in a pilot test recruiting 20 adult patients with chronic diseases (mean age: 66.9 years; SD = 3.43 years), 57.9% of whom were women. Patients were individually asked about any difficulties completing the test and understanding the items and suggestions were solicited. Specific alterations were made to four items.

### Participants’ characteristics

The study recruited 301 adults with chronic disease (mean age: 63.5 years; SD = 15.7) (Supplementary Table S4).

### Content validity

The exploratory factor analysis results revealed a three-factor structure accounting for 47% of the variance (20, 15, and 12% loadings in factors 1, 2, and 3, respectively) (Table 2).

**Table 2:** Death Anxiety Scale (DAS) factor structure

Items	F1	F2	F3	u2
1. I am very much afraid to die.	**-0.73**	0.37	-0.14	0.33
2. The thought of death seldom enters my mind.	**0.41**	-0.09	0.02	0.81
3. It doesn´t make me nervous when people talk about death.	**0.68**	0.01	-0.02	0.53
4. I dread to think about having to have an operation.	0.04	0.09	**0.51**	0.7
5. I am not at all afraid to die.	**0.99**	0.15	0.12	0.14
6. I am not particularly afraid of getting cancer.	0.05	-0.05	-0.27	0.9
7. The thought of death never bothers me.	**0.83**	-0.04	0.02	0.3
8. I am often distressed by the way time flies so very rapidly.	0.05	**0.9**	0.04	0.18
9. I fear dying a painful death.	-0.02	0.09	**0.71**	0.41
10. The subject of life after death troubles me greatly.	-0.17	**0.45**	0.12	0.65
11. I am really scared of having a heart attack.	-0.16	0.3	**0.33**	0.61
12. I often think about how short life really is.	0.00	**0.77**	0.02	0.39
13. I shudder when I hear people talking about a World War III.	0.22	0.26	**0.45**	0.68
14. The sight of a dead body is horrifying to me.	0.03	-0.1	**0.62**	0.68
15. I feel that the future holds nothing for me to fear.	0.33	**0.34**	-0.33	0.73

Bold type denotes higher factor loadings in each factor.

u2 = uniqueness.

Analysis using a principal factor solution with varimax rotation.

As seen in Supplementary Table S4, factor 1 is associated with death-related emotional (DRE) aspects, measured by five items (1, 2, 3, 5, and 7), whereas factor 2 deals with aspects indirectly related to death (IRD), measured by four items (8, 10, 12, and 15). Factor 3 is more heterogeneous, being related to health problems and witnessing death (HWD), measured by five items (4, 9, 11, 13, and 14). Item 6 (“I am not particularly afraid of getting cancer”) had no suitable factor loading in any of the three domains and showed the highest uniqueness value (u2 = 0.9).

**Figure 1:**
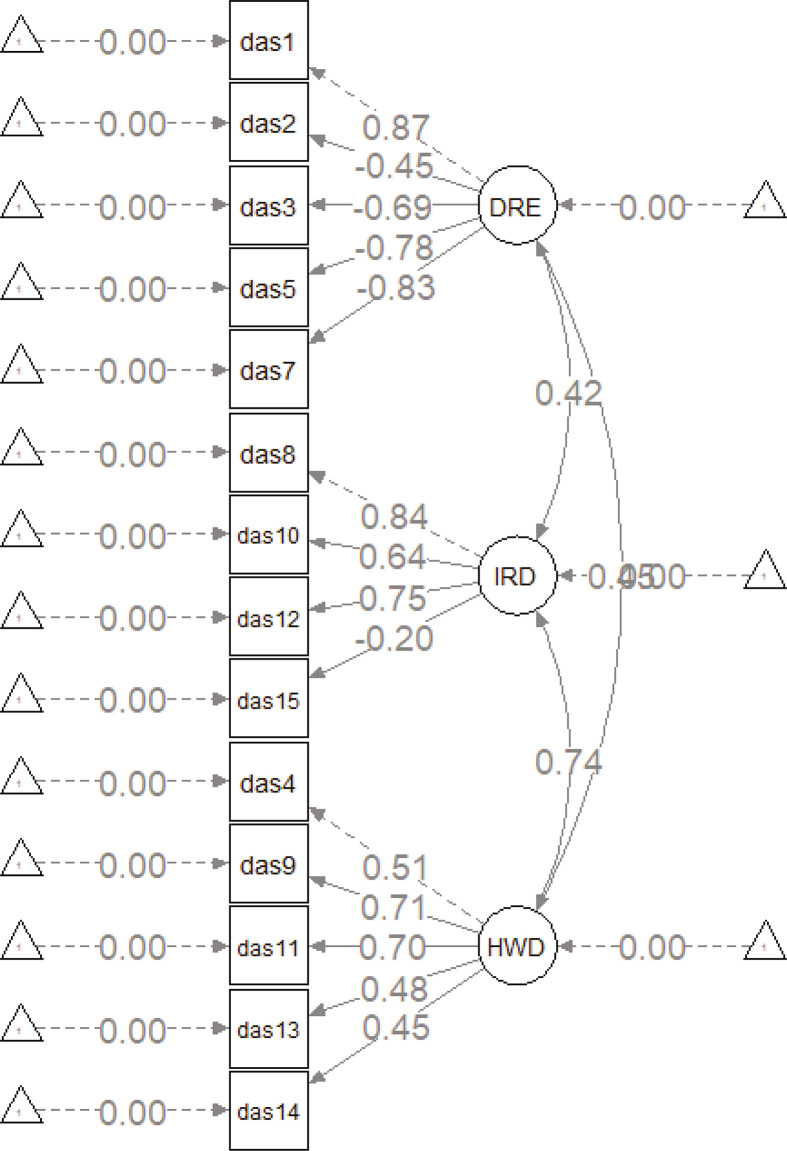
Death Anxiety Scale structural equation system. IRD = indirectly related to death; DRE = death-related emotional; HWD = health problems and witnessing death.

Owing to the dichotomous nature of the items, confirmatory factor analysis was performed using the robust weighted least squares estimation method (Figure 1).

The circles in Figure 1 represent the three domains or factors, rectangles stand for the 14 items, and arrows pointing in a single direction indicate the causal relations between the domain and the item, while the correlations between factors are represented by two-way arrows. The structural equation model constructed had the following estimators: χ^2^ = 135.693, df = 74.000, χ^2^/df = 1.83, RMSEA = 0.053, NNFI = 0.936, CFI = 0.948, GFI = 0.93, and SRMR = 0.108. The values of these indicators demonstrate appropriate fit of the structural model.

### Internal consistency

The reliability estimators obtained from the analysis based on 301 observations were as follows: Cronbach’s alpha: 0.71, McDonald’s omega: 0.76, Guttman’s lambda: 0.74, and greatest lower bound: 0.54. Supplementary Table S3 shows the values of the alpha coefficients and Guttman’s lambda, none of which increased after removing any one of the DAS items.

### Concurrent criterion validity

The study analysis was conducted based on 241 observations, estimating the coefficients for correlations between the total scores of the DAS and DAI scales and between the DAS domain scores and the total DAI score. The Pearson’s coefficient for the correlation between the total scores of both scales was 0.64 (significantly different from 0). Coefficient values greater than 0.45 were found for the correlations between the DAI and the DAS domains, showing that the total scores of the two instruments had a correlation coefficient of 0.64 (Supplementary Table S5).

### Test-retest reliability

The test-retest reliability analysis was performed based on 82 repeated measurements, separated by an interval of 5-7 days (mean of 5 days, interquartile range = 2 days). The scale scores corresponding to each of the two measurements (test and retest) are shown in Supplementary Table S6.

**Figure 2:**
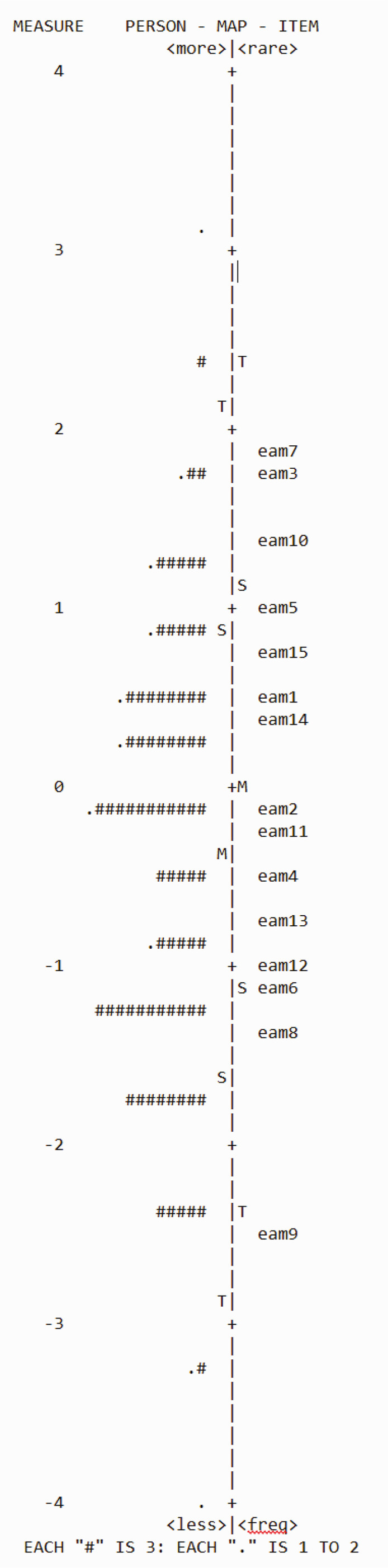
Person-Items map (Wright map). eam = Escala de Ansiedad ante la Muerte; M = Mean; S = 1 standard deviation from the mean; T = 2 standard deviations from the mean.

The stability of the total scores and of the scores corresponding to each of the two domains was estimated using signed rank-sum tests with the two-tailed hypothesis. No statistically significant differences were observed between test and retest measurements, whether for the overall score or any of the domain scores (Figure 2).

As seen in the correlation matrix (Pearson’s correlation coefficients), all correlation values were different from 0 (statistically significantly differentiable 1e, p < 0.00001) (Supplementary Table S7).

Supplementary Table S2 shows Lin’s correlation concordance coefficient values, which indicate an acceptable degree of concordance in domains 1 and 3 and a low degree of concordance in domain 2.

As shown by Bland-Altman goodness-of-fit plots (Supplementary Figures S1, S2, S3, S4, and S5), no correlation patterns were observed based on the different scale scores or in any domain.

### Rasch analysis

The PCAR showed that 34.55% of the variance was explained by the measurement model and that the first residual component had a value of 2.1, equivalent to 9.4% of the total non-explained variance. Moreover, MNSQ infit and outfit values for the items remained between 0.6-1.5. These findings support the hypothesis of unidimensionality. All correlations of standardized residuals had low values (under 0.4), the highest was observed between items 8 and 12. (r = 0.35) (“I am often distressed by the way time flies so very rapidly” – “Often think about how short life really is”). Based on the aforementioned calculations, it can be assumed that the hypothesis of local independence is confirmed.

#### Description of scores in the logit metric scale

The difficulty level of the items was between -2.46 logits and 1.85 logits (mean = 0; SD = 0.16), while the skill level of subjects was between -4.55 logits and 3.17 logits (mean = 0.39; SD = 0.66). Considering the estimates of item difficulty, the item with the least difficulty (equivalent to the item with the smallest discrimination capability and high sensitivity) was item 9 (“I fear dying a painful death”). Item 7 had the greatest difficulty (“The thought of death never bothers me”).

To compare the DAS scores by different groups of variables, the transformed version was used, using logit units on a scale of 0 to 100. Findings are summarized in Supplementary Table S8. None of the differences in means among strata were statistically significant.

#### Measures of item fit

MNSQ infit and outfit statistics all fell within the recommended range (0.5-1.5), indicating adequate fit to the Rasch model.^26^

#### Measures of persons fit

According to some authors,^27^ outfit values standardized to a z-score (ZSTD) > 3 are indicative of poor adjustment in persons. According to that criterion, only two individuals (0.83%) showed poor fit to the Rasch model (Figure 2). Means are close to 0 for both persons and items and have similar variability.

No overlapping items were found. The item with the least difficulty “I fear dying a painful death,” was approximately at a 1 logit distance from the next item; to properly cover the construct, it might be necessary to add items that cover the lower spectrum of difficulty in the scale (e.g., the distance between das8 and das9).

#### Reliability of persons and items

The index of reliability was 0.71 and 0.98 for persons and items respectively. The separation index was 1.56 for persons and 7.2 for items. These findings suggest an adequate level of reliability for both persons and items.

## Discussion

Recognition of DA as an entity that adds to the clinical picture of a chronic patient is essential for clinical follow-up and comprehensive care interventions. DA exacerbates the negative affect of chronic disease, distorts perceptions about recovery, distances patients from health professionals,^17,28,29^ significantly decreases the quality of life of both the patient and the patient’s partner,^17,30^ increases vulnerability to psychological stress and physical and emotional distress,^31^ is predictive of the onset of psychopathologies, and increases the number of hospitalizations and the need for or resistance to pharmacological treatment.^32^ Some authors believe that DA should be studied as a transdiagnostic construct, as it promotes development and maintenance of various mental disorders.^31,32^

Our results show that the cross-culturally adapted version of the DAS in Colombian Spanish comprises three factors that measure DRE, IRD, and HWD. The instrument’s internal consistency is adequate, based on a Cronbach’s alpha of 0.71, which differs from the study by Rivera-Ledesma and Montero-López^33^ conducted in Mexico with older adults (n = 165) and university students (n = 149) (0.86 in older adults and 0.83 in students).^33^ In this regard, the following points were considered:

1. Unlike our study population, some older adults in the Mexican sample may have had a chronic condition but not the entire cohort.
2. The difference in sociocultural context and the meaning of death. In fact, when comparing the results of both studies with a different Spanish version (from Mexico and Spain) against ours, it is clear that, as reported by Sharif et al.,^1^ the psychometric variability in the results is caused by cultural and linguistic differences. This aspect fully justifies the need to further investigate the behavior of the psychometric properties of the instruments applied in different contexts.
3. Variations in the response scale. In our study, analyses were conducted based on the dichotomous scale used in the original instrument, whereas in the study by Rivera-Ledesma and Montero-López,^33^ the dichotomous format was replaced by a Likert-type scale.

Our study found a correlation coefficient of 0.64 between the DAS domain scores and the DAI total score, showing adequate validity in terms of the concurrent criterion between tests. No significant differences were found between the test and retest, whether for the total score or any of the domain scores.

The results of this study confirm the need to always carry out cross-cultural adaptation and analysis of the psychometric properties of instruments that are to be used for research and clinical purposes. It would be unwise to assume that the language used guarantees the reliability and validity of an instrument and understanding by the population.

In addition to an acceptable level of reliability, this Colombian Spanish version of the DAS was shown to be valid, which allows us to conclude that it is a short and effective instrument available for measuring a variable of widespread interest for both research into DA and clinical interventions in chronic adult patients.

The key findings of the Rasch analysis were that the basic assumptions for the model (unidimensionality and local independence) were fully met in the data analysis and that the item “The thought of death never bothers me” had the highest specificity, i.e. it is one that, when responded negatively, was only answered by subjects with high levels of DA.

Moreover, no significant differences in scores were found between subjects from different socioeconomic strata. Items and subjects were adequately fitted to the Rasch model, variability amongst items and subjects was similar, and no redundant items were identified. There was a non-measured space between items das8 and das9. If this space were to be filled, better metrics and properties could be yielded upon analysis. Reliability markers are present.

With regard to the limitations of this study, it is worth mentioning that the sample was limited to chronic patients from a hospital in Bogotá. Colombia is a country with broad cultural and regional diversity. The study sample included adults from both rural and urban areas of the country, but their origin was not specified. Additionally, adult chronic patients from high socioeconomic strata were not represented in this study. Future research should observe the performance of the DAS in different regions of the country and in populations from high socioeconomic strata, even though the latter account for a low percentage of the Colombian population.

Furthermore, psychometric studies must be conducted with the general population and health professionals and all these results should be analyzed as a whole. It is likely that many interesting insights will emerge from comparisons between these three populations. Our research group is currently engaged in studies with these characteristics.
