# Supplementary material for: Psychometric properties of the Death Anxiety Scale for adult chronic patients
Source: Trends Psychiatry Psychother. 2025 Feb 27;47:e20230630. doi: 10.47626/2237-6089-2023-0630 (PMC12611330; doi:10.47626/2237-6089-2023-0630)

Supplementary Table S1 - Factorial structure of Templer's Death Anxiety Scale (DAS)

Authors	Population	Factors
Warren and Chopra[34]	Australian students	Death anxiety<br>General concerns<br>Fear of pain and surgery
Devins[35]	General population (17-97 years old) (n = 211)	Fear of one's death<br>Concern about suffering and waiting too long before dying<br>Subjective nearness to death<br>Death-related fears<br>Disturbing thoughts about death
Lonetto et al.[36]	Professionals with different levels of experience treating dying patients and death	Cognitive affective changes<br>Physical changes<br>Sense of time<br>Stress and pain
Martin[37]	Canadian nurses	Denial of death anxiety<br>General death anxiety<br>Anticipatory fear of death<br>Fear of physical death<br>Fear of a catastrophic death
Lonetto & Templer[38]	Not reported	Four universal factors:<br>Cognitive and affective responses to death<br>Real and/or imaginary physical changes accompanying severe disease and death<br>Perception of the passing of time that may reduce the future and extend the past<br>Pain and stress, either real and/or anticipated, which may arise due to both chronic and terminal illnesses or because of personal fears.
Abdel-Khalek et al.[39]	Egyptian students (n = 428)	Thoughts about death<br>Fear of death<br>Concerns about death<br>Brevity of life<br>Apprehension about the future
Saggino and Kline[40]	Italian general population (n = 257)	Fear of death and dying<br>Passage of time<br>Fear of pain and surgery
Hoogstraten et al.[41]	Dutch general population	Fear of dying in the future<br>Perception of the passing of time<br>The third and four factors are ambiguous<br>The fifth factor is only represented by item 11

Tomás-Sábado and Gómez-Benito[19]	Spanish psychology students (n = 187)	Cognitive–affective factors<br>Pain and disease<br>Death-related stimuli<br>Perception of the passing of time
Rivera-Ledesma and Montero-Lopez[20]	Mexican patients with chronic renal failure	Two factors that were not reported

Table was prepared by the authors.

References not in the main article
34. Warren WG, Chopra PN. Some reliability and validity considerations on Australian data from the death anxiety scale. Omega (Westport). 1979;9:293-9.
35. Devins GM. Death anxiety and voluntary passive euthanasia: Influences of proximity to death and experiences with death in important other persons. J Consult Clin Psychol. 1979;47:301-9.
36. Lonetto R, Fleming S, Mercer GW. The structure of death anxiety: a factor analytic study. J Pers Assess. 1979;43:388-92.
37. Martin T O. Death anxiety and social desirability among nurses. Omega (Westport). 1982;1351-8.
38. Lonetto R, Templer DI. Death anxiety. Washington, D.C: Hemisphere Publishing Corporation; 1986.
39. Abdel-Khalek A, Beshai JA, Templer DI. The structure of Templer's death anxiety scale among Egyptian students. Psychol Rep. 1993;72:920-2.
40. Saggino A, Kline P. Item factor analysis of the Italian version of the death anxiety scale. J Clin Psychol. 1996;52:329-33.
41. Hoogstraten J, Koele P, Van Der Laan J. Templer's death anxiety scale revisited: the Dutch version. Percept Mot Skills. 1998;87:1259-64.

References from the main article
19. Tomás-Sábado J, Gómez-Benito J. Psychometric properties of the Spanish form of Templer's death anxiety scale. Psychol Rep. 2002;91:1116-20.
20. Rivera-Ledesma A, Montero-Lopez Lena M. Propiedades psicométricas de la escala de ansiedad ante la muerte de Templer en sujetos mexicanos. Perspect Psicol. 2010;30:135.

Supplementary Table S2 - Measures of internal consistency after removing each item

Items	raw_alpha	G6
DAS 1-	0.67	0.7
DAS 2-	0.7	0.73
DAS 3-	0.69	0.72
DAS 4	0.69	0.73
DAS 5-	0.69	0.71
DAS 6-	0.71	0.74
DAS 7-	0.69	0.71
DAS 8	0.68	0.71
DAS 9	0.69	0.72
DAS 10	0.69	0.72
DAS 11	0.68	0.71
DAS 12	0.69	0.71
DAS 13	0.7	0.73
DAS 14	0.7	0.73
DAS 15	0.71	0.73

DAS = Death Anxiety Scale; G6 = Guttman's lambda 6.

Supplementary Table S3 - Lin's concordance correlation coefficient

	Lin's ρ	95%CI	Bias correction factor
Total pre-total post	0.8	0.73-0.88	0.99
Domain 1 pre-post	0.72	0.61-0.82	0.99
Domain 2 pre-post	0.62	0.48-0.75	0.99
Domain 3 pre-post	0.7	0.58-0.81	0.99

95%CI = 95% confidence interval.

Supplementary Table S4 - Participants' characteristics

Characteristics	Ratio (%)
Clinical diagnosis	
Cancer	29.2
Cardiovascular diseases	29.2
Respiratory diseases	18.3
Diabetes mellitus	13.0
Renal disease	3.0
Rheumatic or autoimmune disorders	1.7
HIV/AIDS	0.77
Comorbidities	
Organic	88.3
Psychiatric	7.3
Anxiety disorders	23.8
Sleep disorders	23.8

Depression	19.0
Addictions	14.3
Sex	
Women	53.0
Men	47.0
Civil status	
Married or cohabiting	55.0
Single	21.3
Widow/widower	15.7
Inpatient	66.8
Outpatient	33.2
Origin	
Urban	93.0
Rural	7.0
Socioeconomic status	
Low	54.0
Middle	41.0
High	5.1.0
Level of education	
Incomplete primary or secondary school	48.0
High school diploma	25.0
Technical level	12.5
University degree	8.8
Postgraduate degree	2.4
None	3.0
Occupationally active	74.4
Retired	38.0
Requiring assistance by a caregiver	46.0
Supported by family caregivers	97.0
Living with their immediate family	54.0
Catholic religion	83.5
Religious practice	78.2

HIV/AIDS = human immunodeficiency virus/acquired immunodeficiency syndrome.

Supplementary Table S5 - Concurrent criterion validity between Death Anxiety Inventory (DAI) and Death Anxiety Scale (DAS)

	Total DAS	Total DAI	Total F1	Total F2	Total F3
Total DAS	1				
Total DAI	0.6448	1			
Total F1	0.7001	0.4546	1		
Total F2	0.7338	0.4546	0.3078	1	
Total F3	0.7686	0.5427	0.2373	0.4510	1

Supplementary Table S6 - Scores of the pre- and post-measures obtained on Death Anxiety Scale (DAS)

	Mean	SD
Total pre-DAS	6.268293	2.998143
Total post-DAS	6.219512	3.220399
Total Domain 1_Pre	1.182927	1.388916
Total Domain 1_Post	1.365854	1.45307
Total Domain 2_Pre	1.902439	1.117866
Total Domain 2_Post	1.865854	1.108466
Total Domain 3_Pre	2.54878	1.371243
Total Domain 3_Post	2.414634	1.456795

SD = standard deviation.

Supplementary Table S7 - Correlation coefficients between pre- and post-Death Anxiety Scale (DAS) measurements

	Total pre	Total post	F1 pre	F2 pre	F3 pre	F1 post	F2 post	F3 post
Total pre	1.00							
Total post	**0.8058**	1.00						
p-value	0.0000							
F1 pre	0.7204	0.6478	1.00					
p-value	0.0000	0.0000						
F1 post	0.6035	0.7741	**0.7250**	1.00				
p-value	0.0000	0.0000	0.0000					
F2 pre	0.7667	0.5650	0.3933	0.2959	1.00			
p-value	0.0000	0.0000	0.0003	0.0070				
F2 post	0.5645	0.7139	0.3690	0.3681	**0.6170**	1.00		
p-value	0.0000	0.0000	0.0000	0.0007	0.0000			
F3 pre	0.7235	0.5874	0.2124	0.3007	0.4058	0.3333	1.00	
p-value	0.0000	0.0000	0.0554	0.0060	0.0002	0.0022		
F3 post	0.6469	0.7619	0.3587	0.3415	0.4118	0.3942	**0.7005**	1.00
p-value	0.0000	0.0000	0.0009	0.0017	0.0001	0.0002	0.0000	

Supplementary Table S8 - Distribution of Death Anxiety Scale (DAS) scores

Variable	Obs.	Mean	SD
Gender			
Female	131	47.08374	13.48422
Male	108	44.67796	14.61005
Setting			
Rural	13	50.04385	18.71211
Urban	227	45.94084	13.71313
Practices a specific religion or faith			
No	52	45.85769	13.87961
Yes	183	46.53153	13.84423
Socioeconomic strata			
1	23	47.54957	14.74498
2	106	46.57189	14.39278
3	97	44.78691	13.52415
4	13	49.71154	14.71616
Relationship status			
Single	51	47.19922	14.04423
Married	98	45.3299	13.55493
In a relationship, cohabiting	35	45.78686	15.84817
Widower	38	47.03132	13.69782
Separated/divorced	18	45.02278	14.52257
Educational level			
None	7	40.51286	15.48408
Incomplete elementary	20	48.8415	14.64996
Elementary	77	45.81481	13.37998
Incomplete high school	13	45.89462	11.33481
High school	62	48.63968	14.0545
Technical degree	32	43.52156	12.62556
Undergraduate	22	44.90727	17.72187
Graduate	5	35.334	12.22989
Lives alone			
No	217	46.33544	14.25649
Yes	24	43.97333	11.63695

SD = standard deviation.

Supplementary Figure S1 - Total Death Anxiety Scale (DAS) scores and scores by domain.

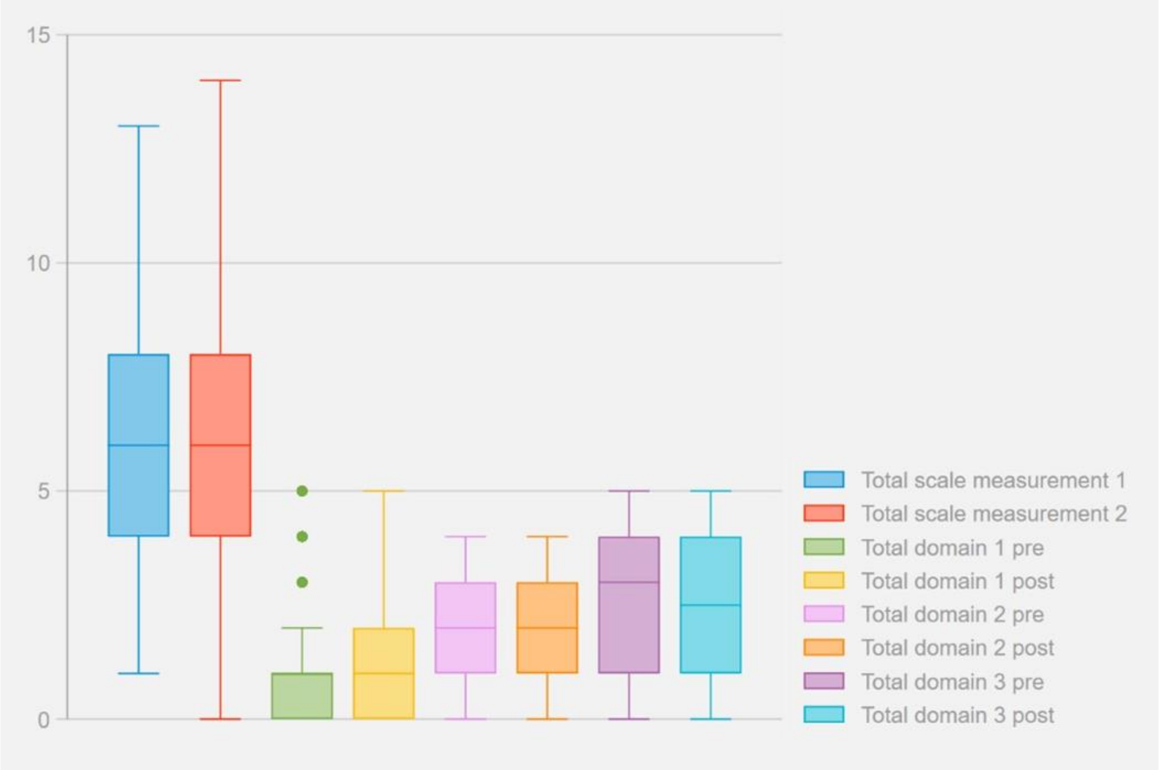

Supplementary Figure S2 - Bland-Altman Limits of agreement of Death Anxiety Scale (DAS): pre- and post-total score measurements.

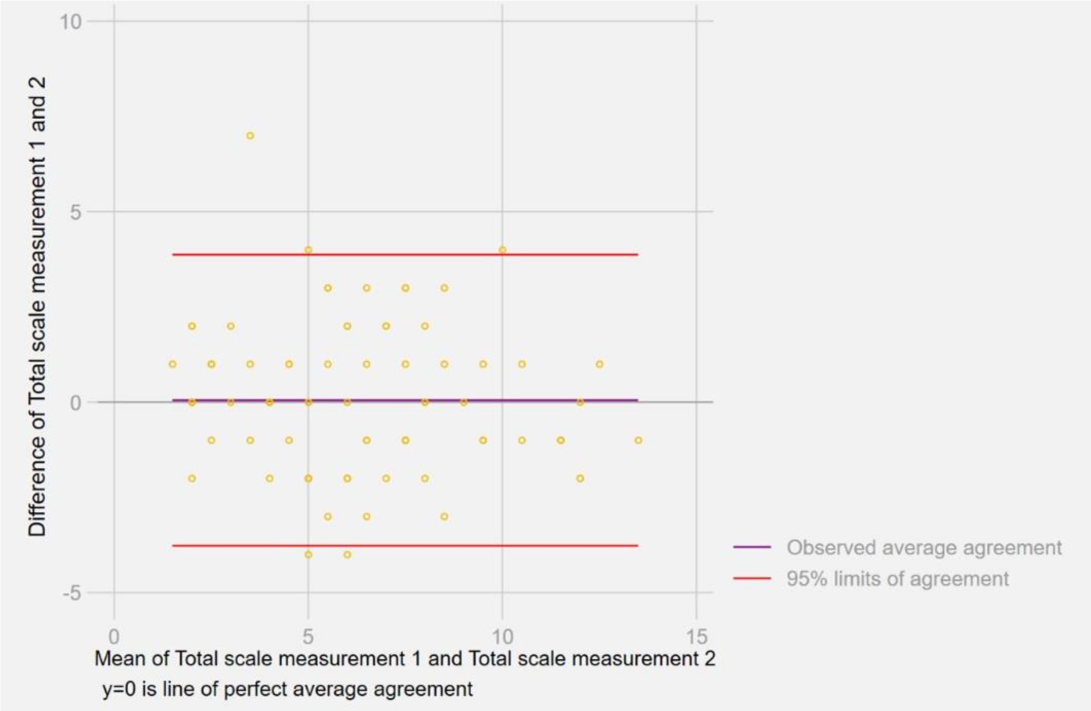

Supplementary Figure S3 - Bland-Altman limits of agreement in domain 1: pre- and post-measurements.

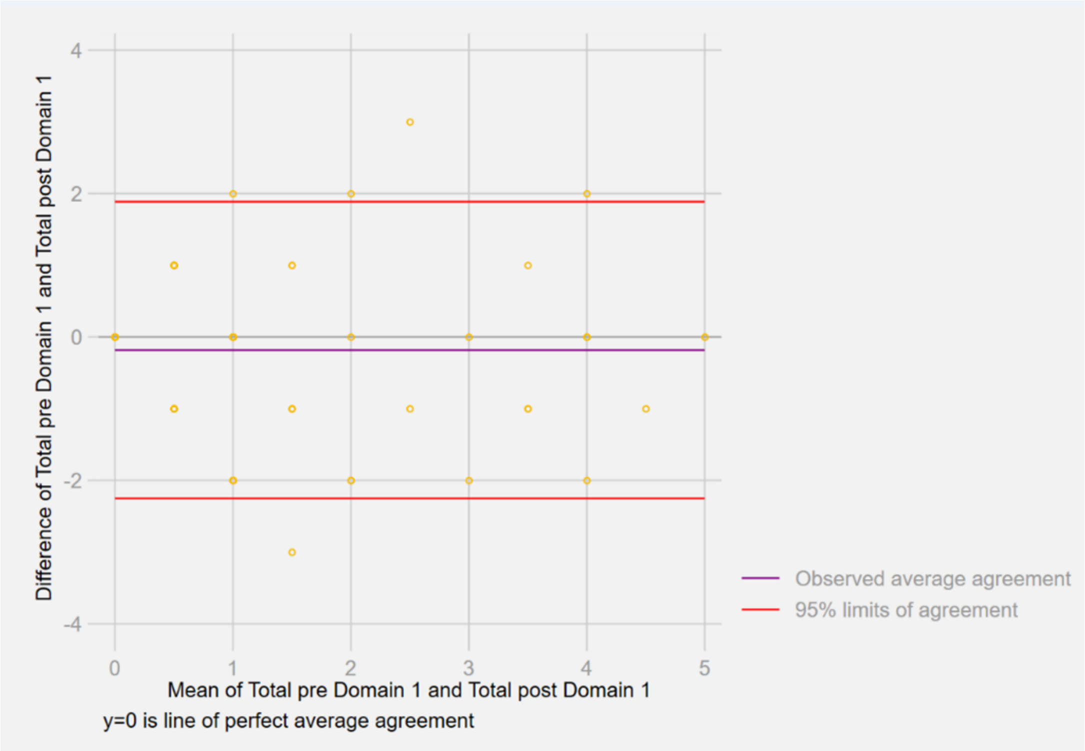

Supplementary Figure S4 - Bland-Altman limits of agreement in domain 2: pre- and post-measurements.

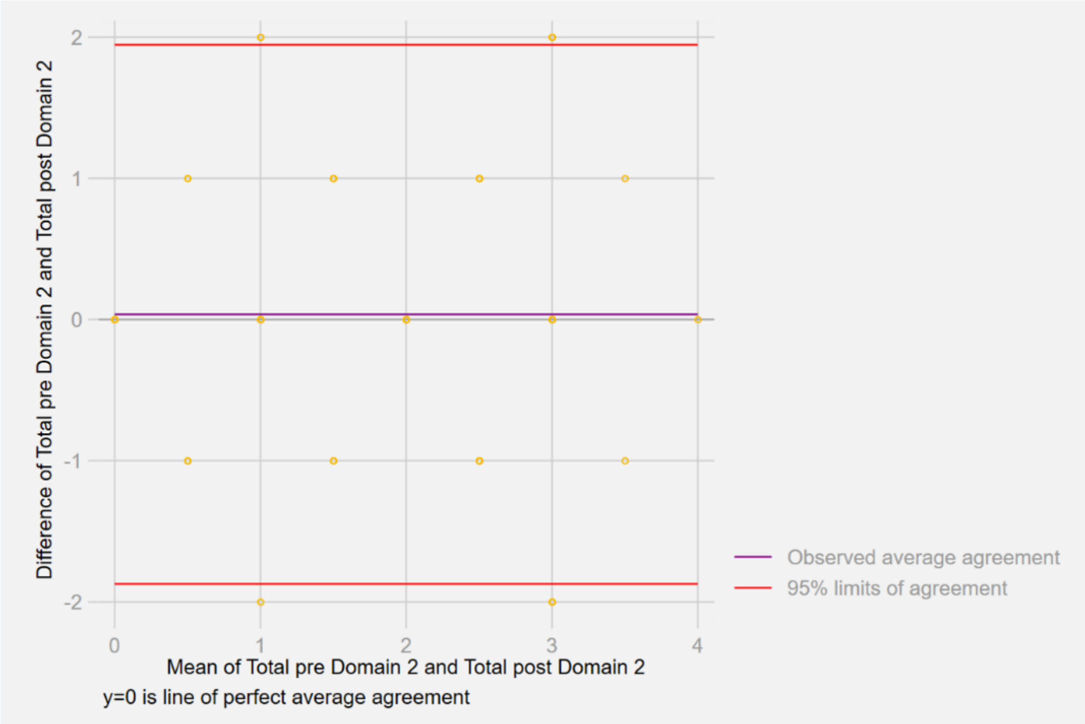

Supplementary Figure S5 - Bland-Altman limits of agreement in domain 3: pre- and post-measurements.

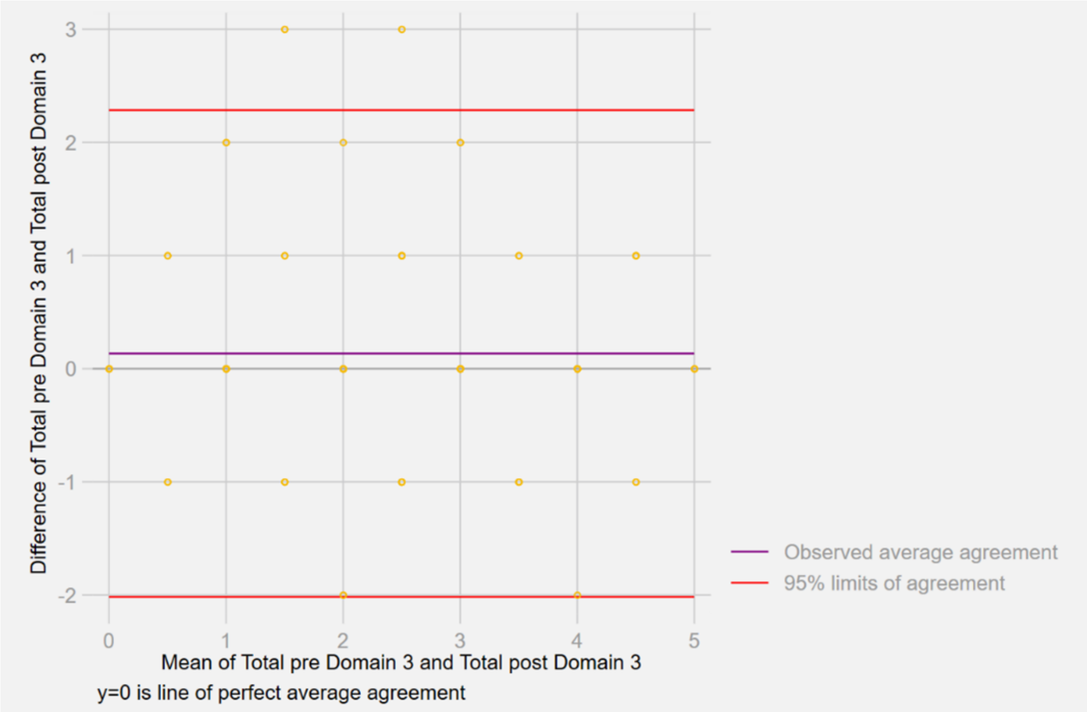

Supplement: Supplementary file 1 [file 2238-0019-trends-47-e20230630_suppl01.pdf]
